# You won’t guess that: On the limited benefits of guessing when learning a foreign language

**DOI:** 10.3758/s13421-021-01254-2

**Published:** 2021-12-15

**Authors:** Ewa Butowska, Maciej Hanczakowski, Katarzyna Zawadzka

**Affiliations:** 1 Interdisciplinary Center for Applied Cognitive Studies, SWPS University of Social Sciences and Humanities, ul. Chodakowska 19/31, 03-815 Warsaw, Poland; 2 Faculty of Psychology and Cognitive Studies, Adam Mickiewicz University, Poznań, Poland

**Keywords:** Guessing, Restudy, Item memory, Associative memory, Education

## Abstract

Guessing the meaning of a foreign word before being presented with the right answer benefits recognition performance for the translation compared to reading the full translation outright. However, guessing does not increase memory for the foreign-word-to-translation associations, which is crucial for language acquisition. In this study, we aimed to investigate whether this disadvantage of guessing for performance in cued-recall tests would be eliminated if a restudy phase was added. In Experiments 1–3, we consistently demonstrated that guessing resulted in lower cued-recall performance compared to reading, both before and after restudy. Even for items for which participants successfully recalled their initial guesses on the cued-recall test, accuracy levels did not exceed those from the reading condition. In Experiment 4, we aimed to generalize our findings concerning restudy to a different set of materials – weakly associated word pairs. Even though this time guessing led to better performance than reading, consistent with previous studies, this guessing benefit was not moderated by adding a restudy phase. Our results thus underscore the importance of the initial learning phase for future learning and retention, while undermining the usefulness of the learning-through-guessing strategy for acquiring foreign language vocabulary.

## Introduction

A question of how to optimize one’s learning has been of interest to researchers for decades (e.g., Atkinson, 1972). One learning technique that holds promise of making learning more effective is attempting to guess the answer to a question before being provided with the correct answer in the form of feedback. Kornell et al. (2009) developed a paradigm, using lists of related word pairs as study materials, that demonstrates the effectiveness of guessing in supporting learning. Participants are asked either to read and learn the full word pair, or are first presented with the first word only and asked to guess what the second word might be before being presented with that word. The results suggest that guessing, which almost always leads to errors, is superior to reading in terms of the final cued-recall test performance (see also Knight et al., 2012; Kornell et al., 2009; Vaughn & Rawson, 2012; see Kornell, 2014, for similar findings using trivia questions as learning materials).

While the basic paradigm of Kornell et al. (2009) reveals robust and replicable benefits of guessing, a number of boundary conditions for this effect have also been described. One of these concerns the relationship within the studied material. For guessing to benefit memory, the target word must be conceptually related to the cue, at least when a memory test relying on cue-target associations is used. No benefits – or even costs – of guessing are observed when unrelated word pairs are learned (Bridger & Mecklinger, 2014; Grimaldi & Karpicke, 2012; Huelser & Metcalfe, 2012; Seabrooke et al., 2021). However, an interesting exception to the usual pattern of no benefits of guessing when words within a pair are not semantic associates has been demonstrated by Potts and Shanks (2014; see also Potts et al., 2019). In their study, participants learned Euskara-English translations as well as rare English words paired with their more common meanings. Although in this case there was the strongest possible semantic relationship between cues and targets – they were the same – this relationship was not available to participants who were assumed to be generally unfamiliar with the cue words. Under these conditions, Potts and Shanks did find benefits of guessing, as performance in a final multiple-choice test was higher in the guessing than in the reading condition. This allowed the authors to conclude that “generating errors could be helpful to memory even during the learning of novel material” (p. 662).

The above evidence for guessing benefits when learning foreign words may encourage using this strategy in educational settings. However, more research is needed to understand the mechanisms responsible for this pattern and the possible limitations of this learning strategy, particularly in light of an apparent discrepancy with the results commonly obtained with unrelated pairs of words. One attempt at better understanding of the role of guessing in learning foreign vocabulary has been undertaken by Seabrooke et al. (2019). Like Potts and Shanks (2014), they used rare English words with their common equivalents and Euskara-English pairs in their experiments, but included various test formats: in addition to simple recognition, they also used cued-recall and associative-recognition tests. Although the guessing benefit described by Potts and Shanks was replicated in the simple-recognition test, no such benefit was observed in the two remaining tests. The authors concluded that guessing strengthens both targets and cues, as measured by recognition tests, but not the association between them that would be necessary for correct responding in associative-recognition or cued-recall tests. This effectively means that even if associations become strengthened by guessing when there is a semantic relationship between the two words, there is also some additional mechanism that operates to strengthen cues and targets in separation (see also Zawadzka & Hanczakowski, 2019).

Overall, the studies reviewed above present a somewhat mixed message regarding the utility of guessing as a strategy for learning novel materials such as foreign vocabulary. On the one hand, changes in memory caused by guessing – as detected in simple-recognition tests – seem to support Potts and Shanks’ (2014) argument concerning the usefulness of guessing for learning novel materials. On the other hand – simple recognition does not seem all that crucial as a criterion test for assessing memory in everyday situations. Taking foreign vocabulary acquisition as an example, it is vital to learn what the foreign word means – that is, the cue-to-target association – and merely knowing that a certain word occurred during study is of much less importance. If guessing does not benefit memory for associations, then arguably it should not be promoted as a means of learning foreign vocabulary.

However, the studies reviewed above all suffer from one caveat. In actual educational settings, rarely are any materials studied only once, and so it is crucial to consider how guessing interacts with further attempts to master the to-be-learned materials. There are at least two reasons to suspect that even in cases when guessing does not benefit memory when applied on its own, it could still be beneficial when followed by a restudy session. The first reason is related to numerous observations of beneficial effects of retrieval for new learning, a pattern generally referred to as test-potentiated learning (e.g., Izawa, 1971; Karpicke, 2009). That retrieval augments learning has been demonstrated across a range of materials, which also included foreign vocabulary pairs (Arnold & McDermott, 2013a) of the sort used in guessing studies. Here of particular interest are the results of Arnold and McDermott (2013b), who manipulated the presence of tests after the initial study phase as well as the presence of a subsequent restudy phase. Not only do their findings show that retrieval improves memory, but also that restudy results in greater memory benefits when preceded rather than not preceded by a test. Although tests given after initial study differ from guessing before the presentation of the study materials, an argument can be made that both situations involve retrieval attempts and hence may produce similar patterns of potentiated learning at restudy. Kornell et al. (2015) have argued that what matters for test-potentiated learning is retrieval *attempt* rather than retrieval *success*, in which case unsuccessful guessing may indeed be similar to a test in maximizing the effectiveness of restudy.

The second reason why restudy might moderate the influence of guessing on memory stems from recent work on the relationship between memory for items and associations. This work suggests that learning associations may be determined by memory for individual to-be-associated items. For example, Reder et al. (2016) demonstrated that memory for associations between a pair of Chinese characters depends on the familiarity of individual components of these associations. When individual components are made more familiar due to their repeated training, associations between them can be more effectively encoded. The work on source memory similarly shows that associating items with their sources is more effective when items themselves are made more familiar by being primed (Gagnepain et al., 2008; but see Kim et al., 2012). Such findings map onto a proposal by Popov and Reder (2020), who postulated the existence of encoding resources that can be depleted and then restored by the passage of time. In this formulation, when items are more familiar, fewer resources are required for their encoding and the remaining resources can be spent on encoding associations, supporting contextual memory. Adapting the encoding-resources hypothesis to the issue of learning novel materials through guessing starts with the observation that guessing improves memory for individual components of the to-be-learned pairs (Seabrooke et al., 2019). This improvement in memory for individual cues and targets may result in facilitated encoding of associations at restudy. In other words, translations of foreign language words, once strengthened by guessing, could be then easier to bind with their foreign counterparts, which would show as an advantage on the final cued-recall test.

In order to investigate how restudy affects memory performance in the guessing paradigm, in Experiments 1–3 we had participants complete a study phase for Finnish-Polish word pairs, with half of the pairs requiring guessing the translation and the other half being presented intact. This initial study phase was then followed by a restudy phase and a cued-recall test. After a single presentation, we predicted better test performance in the read condition, replicating previous findings (Seabrooke et al., 2019, Experiment 5). However, we also expected restudy to be more beneficial for items from the guess condition due to stronger memory representations of targets that should be easier to bind with their Finnish counterparts. In effect, we predicted that restudy would either minimize the difference across read and guess conditions, or even reverse it. Ultimately, thus, our purpose was to establish whether the opportunity to restudy novel materials would make initial guessing an equivalent or better learning strategy than reading.

## Experiment 1

In the present experiment, we sought to establish whether restudy changes the pattern of differences across guess and read conditions when learning foreign language vocabulary. We asked our participants to learn Finnish-Polish pairs via either reading or guessing with immediate feedback, and then we manipulated whether these pairs were presented for restudy via reading. We assessed memory both by the means of simple recognition, to confirm that guessing strengthens individual targets, and cued recall, to first confirm that guessing yields costs compared to reading for associative memory, and then establish whether this cost is ameliorated by restudy.

### Method

#### Participants

Sixty students and graduates from Warszawa and Łódź (11 male; age range 18–57 years, mean: 26.4) with fluency in Polish but no previous knowledge of Finnish participated in the experiment in exchange for course credit or gift cards. For all other experiments reported here we attempted to recruit the same number of participants; a post hoc power analysis showed that this sample size resulted in 75% power to detect an interaction of interest of the size obtained in Experiment 1. We excluded two participants as they failed to provide any guesses during the learning phase. This resulted in a final sample of 58 participants. The study was approved by the Department of Psychology Ethics Committee at the SWPS University.

#### Materials and design

**Fig. 1:**
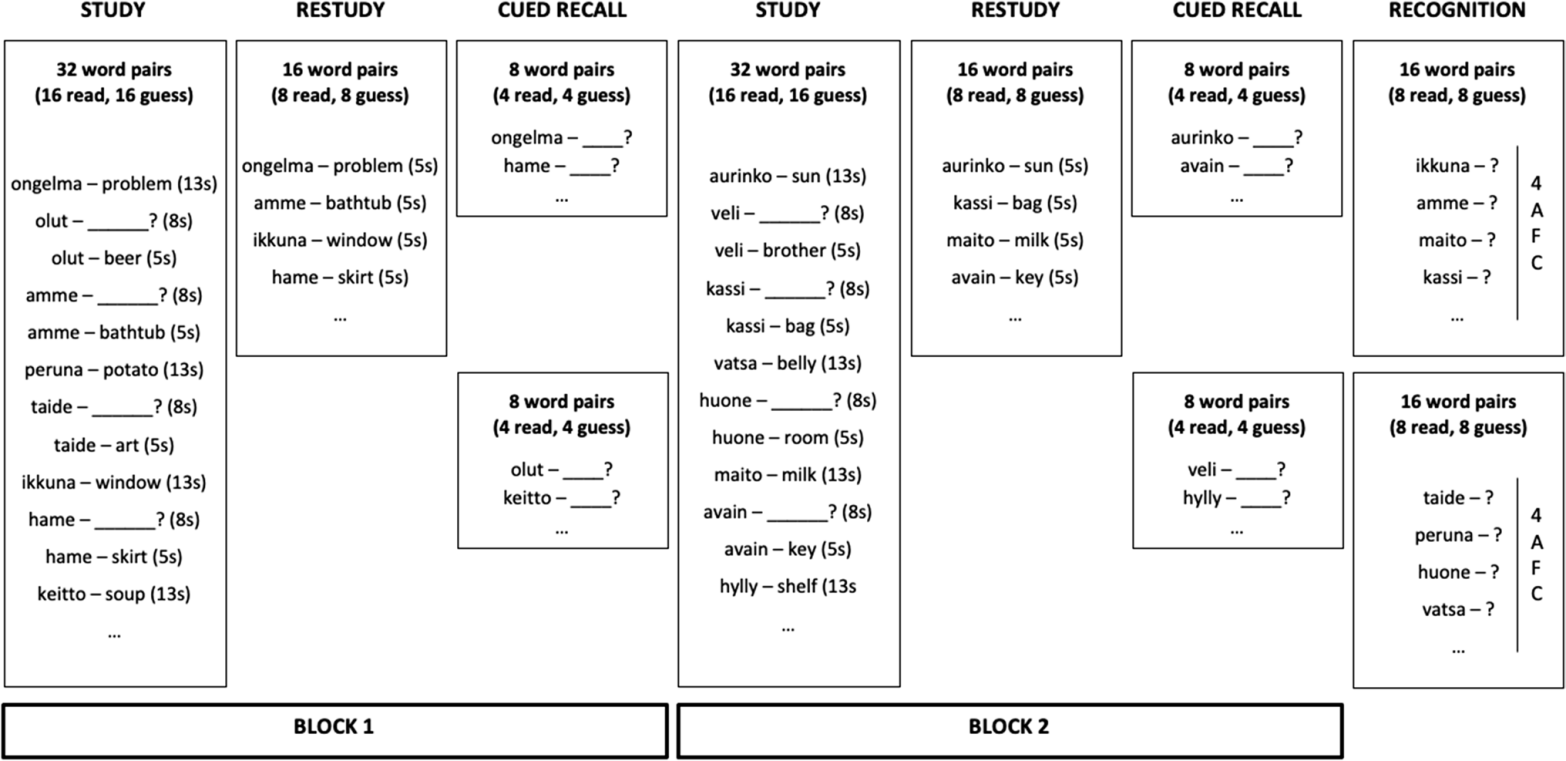
A schematic representation of Experiment 1. For convenience, Finnish-English (rather than Finnish-Polish) translations are presented

Sixty-four pairs of Finnish-Polish nouns were used as study materials, and two additional pairs were used for practice. Finnish words were used as cues and their Polish translations were the targets. As Fig. 1 shows, the study list was divided in two, with each half being assigned to one study-test block. Within each block, 16 words from the study list were assigned to the guess condition, and the remaining 16 to the read condition. In the read condition full word pairs were presented in the middle of the screen. In the guess condition the Finnish cue appeared first and participants had to guess at and type in its Polish meaning. After that, corrective feedback – the Polish translation – was presented.

After the presentation of the 32 pairs, half of them (eight from the read condition and eight from the guess condition) was presented for restudy. The pairs were always presented for restudy in full, and the assignment of words to the restudy versus no-restudy conditions was counterbalanced across participants. The restudy phase was followed by a cued-recall test for 16 of the already presented pairs, four from each condition. Participants were presented with Finnish words as cues and asked to type in their translations. After the first cued-recall test, the second study-test block followed, which was identical to the first block bar the replacement of all materials. After the second cued-recall test, a final four-alternative forced-choice recognition test was administered for the remaining half of the pairs which was not tested yet (16 from each block, eight from each condition). The cue was again the Finnish word, which appeared with the correct translation (the target) and three novel lures presented in a random order. The lures were Polish words with the same number of letters as the target, and none of them was presented in any of the earlier phases of the experiment.

Thus, the study had a 2 (learning condition: guess, read) x 2 (restudy condition: restudy, no restudy) x 2 (test format: cued recall, recognition) within-subject design. The assignment of words to conditions, study-test blocks, and test types, as well as the order of presentation of all items at study and test were counterbalanced across participants.

#### Procedure

Participants were tested individually or in small groups. They were instructed that their task would be to learn Polish translations of Finnish words for a future test. They were then informed that they would encounter two types of study trials in the procedure. On some trials, both words would be presented for study for 13 s, while some other trials would require guessing at the meaning of the presented Finnish word within 8 s before being presented with the correct translation for five seconds. Participants were also informed that afterwards they would see some of the pairs presented for a second time for study. After these initial instructions, participants underwent a training phase consisting of studying and being tested on two pairs (one from each learning condition). Then, participants completed the first study phase followed by a restudy phase. In the restudy phase all pairs were presented for five seconds for participants to read. The restudy phase was followed by instructions for the first self-paced cued-recall test, which underscored that participants had to retrieve the correct translations of the Finnish words and not their own guesses. The cued-recall test was followed by the second study-test block. After the second cued-recall test, participants were given a final self-paced recognition test for pairs from both blocks that were not tested before.

### Results and discussion

During the learning phase, nine participants correctly guessed the meaning of a single Finnish word (sisko – sister). This resulted in an average of 0.5% correctly guessed items, which were removed from subsequent analyses. We performed an additional control analysis excluding the trials on which our participants failed to provide their guess for Experiments 1, 2, and 3. However, no difference was observed compared to analyses including the trials with no typed guesses. For simplicity, here and in the subsequent experiments we report the analyses which include both typed guesses and questions left blank throughout the experiment, unless stated otherwise.^1^

#### Recognition

Table 1 presents performance on the multiple-choice test across conditions. We performed a repeated-measures analysis of variance (ANOVA) with learning condition (read, guess) and restudy opportunity (restudy, no restudy) as factors. This revealed a significant main effect of learning condition, *F*(1,57) = 7.87, *p* = .007, η_p_^2^ = .12. Overall, guessing (*M* = .94, *SD* = .09) benefitted final recognition performance to a greater extent than reading (*M* = .90, *SD* = .14). Also, there was a significant main effect of restudy, *F*(1,57) = 31.61, *p* < .001, η_p_^2^ = .36. Restudied items were better remembered, *M* = .95, *SD* = .09, compared to *M* = .89, *SD* = .14, for non-restudied items. Finally, there was a significant interaction between the two factors, *F*(1,57) = 6.01, *p* = .017, η_p_^2^ = .09. This interaction arose because there was a significant difference between the two learning conditions when no restudy opportunity was available, *t*(57) = 3.09, *p* = .003, *d* = 0.41; however, this difference was no longer significant for pairs that were restudied, *t*(57) = 0.82, *p* = .417, *d* = 0.11. Still, as the results after restudy were near ceiling, we refrain from interpreting this interaction.

**Table 1:** Recognition performance as a function of learning condition and restudy opportunity in Experiment 1

	Learning condition	
Restudy opportunity	Guess	Read
No Restudy	.92 (.01)	.86 (.02)
Restudy	.96 (.01)	.95 (.01)

*Note.* Standard errors of the mean are given in parentheses

Together, the significant main effect of learning condition and, more specifically, the difference in recognition performance between the read and guess conditions with no restudy, demonstrate that target memory was improved after attempting to guess the target compared to merely reading the cue-target pair. This is consistent with previous findings (Potts & Shanks, 2014; Seabrooke et al., 2019), and supports our assumption that guessing leads to strengthening of targets. This allowed us to investigate whether greater target strength translates into better encoding of associations at restudy, as measured by the cued-recall test.

#### Cued recall

Table 2 presents mean cued-recall performance across conditions.^2^ A 2 (learning condition: read, guess) x 2 (restudy opportunity: restudy, no restudy) ANOVA revealed a significant main effect of learning condition*, F*(1,57) = 20.54, *p* < .001, η_p_^2^ = .27. Overall, reading benefitted performance to a greater extent than guessing (*M* = .48, *SD* = .32, and *M* = .40, *SD* = .30, respectively). Also, there was a significant main effect of restudy, *F*(1,57) = 264.23, *p* < .001, η_p_^2^ = .82. Restudying the translations resulted in better cued-recall performance compared to not restudying (*M* = .63, *SD* = .25, and *M* = .25, *SD* = .25, respectively). Most importantly, the interaction between the two factors was not significant, *F*(1,57) = 0.25, *p* = .621, η_p_^2^ = .004.

**Table 2:** Cued-recall performance as a function of learning condition and restudy opportunity in Experiments 1–3

	Learning condition	
Experiment and restudy	Guess	Read
Experiment 1		
No restudy	.20 (.03)	.29 (.03)
Restudy	.59 (.03)	.67 (.03)
Experiment 2		
No restudy	.16 (.02)	.20 (.03)
Restudy	.31 (.03)	.39 (.03)
Experiment 3		
No restudy	.17 (.02)	.20 (.03)
Restudy	.33 (.03)	.37 (.03)

*Note.* Standard errors of the mean are given in parentheses

The first experiment failed to show any benefits of guessing in cued recall even after restudying the unfamiliar foreign vocabulary translations. As expected, without restudy, reading was superior to guessing when a cued-recall test was used. However, even after restudy neither was this benefit of reading reversed, nor attenuated. This is despite the fact that the recognition results replicated previous findings of higher effectiveness of guessing compared to reading. This finding confirmed that guessing did enhance target memory in our paradigm, which was crucial for our predictions. Nevertheless, better target memory in this case did not facilitate encoding of cue-to-target associations.

## Experiment 2

The results of Experiment 1 failed to produce any benefits of guessing when foreign language translations were used as study materials, despite the addition of a restudy phase and the fact that targets in the guess condition were better remembered compared to those that were merely read. It is thus prudent to evaluate the procedure used in this experiment to establish why restudy might have failed to produce any memory benefits in the guess compared to the read condition.

One of the main concerns regarding guessing as a learning strategy is producing errors which can potentially interfere with memory for targets. In fact, a whole tradition of errorless approach to learning strategies stands on the above premise (e.g., Jones & Eayrs, 1992). Nevertheless, some recent work suggests that errors may be used as scaffolding for correct retrieval if they are retrieved during test. Metcalfe and Huelser (2020) showed that in the guessing task, performance in the guess condition was higher on trials on which participants could retrieve their original guess compared to those on which the guess could not be accessed. This observation chimes with the work on interference by Wahlheim and Jacoby (2013), demonstrating that the usual effect of proactive interference of studying an AB pair for memory of an AC pair is reversed when participants detect the change of targets when the AC pair is presented, and – crucially – recollect the original AB pair on the final test. Also in this case, thus, a potentially interfering response can serve as a mediator (Pyc & Rawson, 2010) for accessing the correct response, in the same way as errors in the guessing paradigm.

In Experiment 1 we had no way of knowing whether our participants were able to recollect their guesses at retrieval. If these were not retrieved, then they could not serve as self-generated episodic mediators for accessing the correct translations of the Finnish words. Therefore, in Experiment 2 we introduced a direct measure of *guess recollection* at test to assess the affordance of errors as potential episodic mediators. In addition to that, to make our participants think back to the initial learning phase, we also introduced a measure of *guess detection* at restudy by asking participants whether a given pair was studied in the read or guess condition. Such encouragement to retrieve guesses at restudy may allow for their integration with to-be-learned targets, facilitating their use as scaffolding for retrieval at the time of the final test, thus benefiting performance in the guess compared to read condition.

### Method

#### Participants

Sixty university students and graduates (14 male; age range 19–46 years, mean: 28.5) took part in the experiment in exchange for course credit or gift cards. After testing the first 39 participants, we were forced to cease face-to-face data collection due to the Covid-19 pandemic. The remaining 21 participants were thus tested individually via a video link, with constant supervision from the experimenter. We continued with on-line testing for the rest of the experiments reported here.

Two participants had to be excluded due to procedure errors, another three because their final accuracy was close to zero, and another four because they failed to type in any guesses during the learning phase. This gave us a final sample of 51 participants.

#### Materials, design, and procedure

**Fig. 2:**
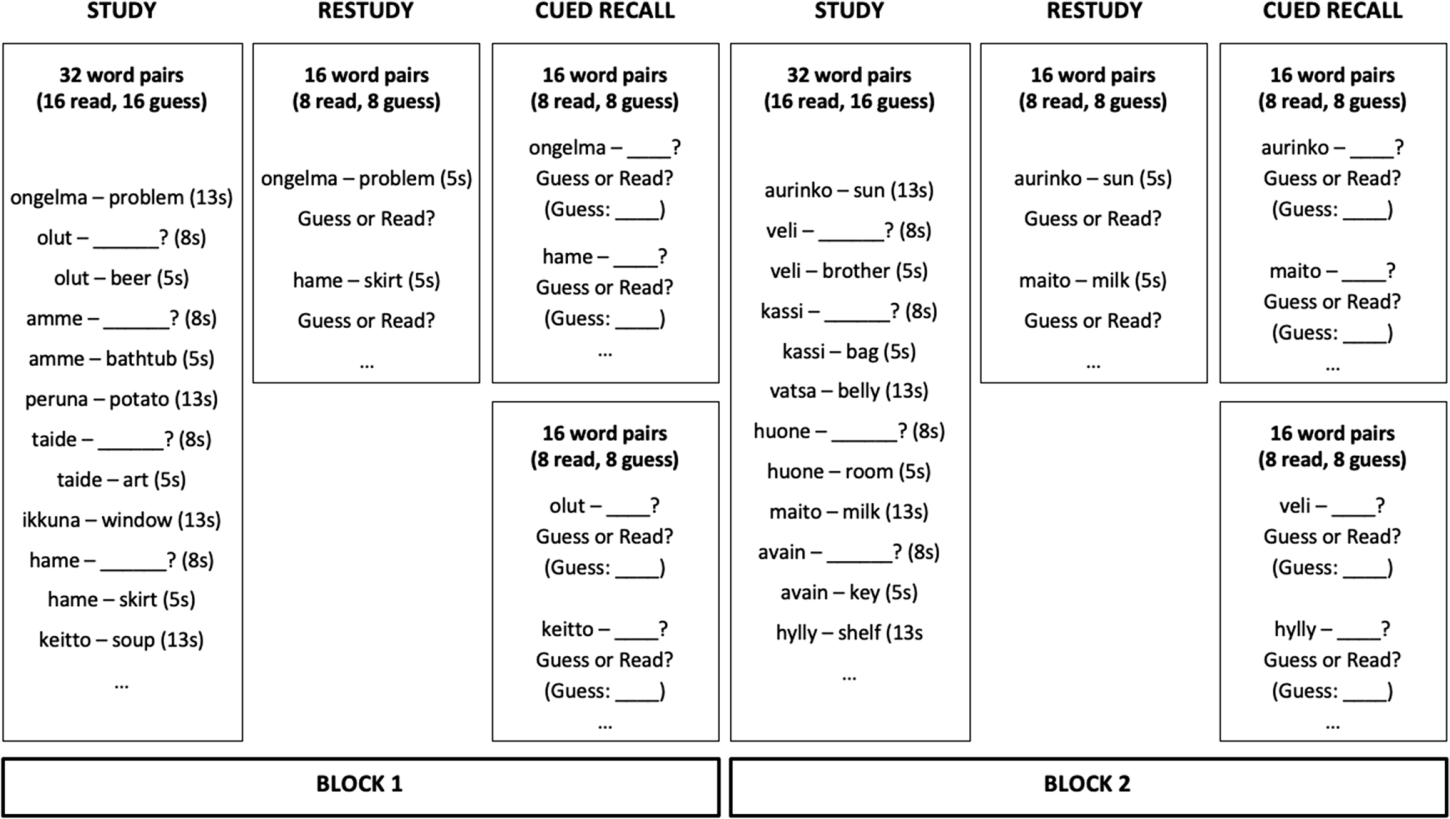
A schematic representation of Experiments 2 and 3. In Experiment 2, guesses were unconstrained, while in Experiment 3 each guess had to begin with the same two letters as the cue. On cued-recall tests participants were also asked to type in their guesses for each pair they believed was studied in the guess condition

The design of Experiment 2 is presented in Fig. 2. The materials and design were the same as in Experiment 1 except for the following changes. In the restudy phase, after the presentation of each pair, a question asking whether this pair was previously presented in the read or guess condition was presented in a forced-choice alternative format, and participants had to choose one of the options in order to advance to the next pair. We will refer to this measure as *guess detection*. Following Metcalfe and Huelser (2020; see also Yan et al., 2014), at test we also asked the same question about the learning condition after each item. If the “guess” option was chosen, a follow-up question appeared, asking to type in the initial guess. This was our direct measure of *guess recollection*. We also eliminated the recognition test from the procedure. This allowed us to have twice as many trials to analyze – that is, we had 32 pairs per each cued-recall test compared to 16 in Experiment 1. Finally, we replaced the often correctly guessed word pair “sisko-sister” with a new one. The laboratory and online versions of the procedure were the same, except for the format and content of the consent form, and the duration of the experiment also did not differ between the versions.

### Results and discussion

During the learning phase, three participants correctly guessed the meaning of a word. This resulted in an average of 0.18 % correctly guessed items, which were removed from subsequent analyses.

#### Cued recall

Table 2 presents mean cued-recall performance across conditions. A repeated-measures 2 (learning condition: guess, read) x 2 (restudy opportunity: restudy, no restudy) ANOVA revealed a significant main effect of learning condition, *F*(1,50) = 11.80, *p* = .001, η_p_^2^ = .19. Overall, reading improved cued-recall performance compared to guessing (*M* = .29, *SD* = .23 and *M* =.23, *SD* = .20, respectively). Also, there was a significant main effect of restudy, *F*(1,50) = 113.36, *p* < .001, η_p_^2^ = .69. Restudying the translations resulted in an overall higher performance of .35 (*SD* = .22) compared to .18 (*SD* = .17) for not restudied items. The interaction between these factors was not significant, *F*(1,50) = 1.77, *p* = .190, η_p_^2^ = .03. These results replicate those of Experiment 1.

Notably, the benefit of restudy was attenuated in the present experiment. In Experiment 1, restudying increased final recall performance on average by .39. Here, this average dropped to .17. This may be due to the introduction of an additional task of guess detection during a restudy phase, which might have increased task load and resulted in a reduced benefit of restudy.

#### Guess detection at restudy

**Fig. 3:**
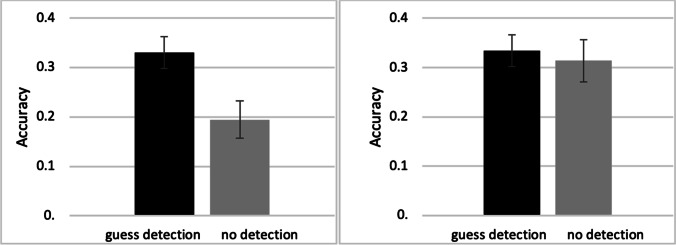
Cued-recall performance in Experiments 2 (**left panel**) and 3 (**right panel**) conditionalized on correct guess detection at restudy. Error bars represent standard errors of the mean

We divided restudied items in the guess condition depending on whether participants correctly classified them as guesses in the restudy phase (i.e., guess detection). Note that this analysis excludes all items from the no-restudy condition. The majority of items (*M* = .72, *SD* = .19) were correctly classified at restudy as being from the guess condition. Cued-recall performance for items with and without guess detection at restudy can be seen in Fig. 3. There was a significant difference in cued-recall performance between items correctly identified as being studied in the guess condition compared to those incorrectly labelled as read, *t*(45) = 2.89, *p* = .006, *d* = 0.43, with an advantage for items with guess detection.^3^

#### Guess recollection at test

The design of Experiment 2 allowed us to compare cued-recall performance for items with and without successful guess recollection at test. In the following analyses we only included items with guesses typed in during the initial study phase, as for those items we could determine whether the guess retrieved at test matched that made at study. This is reflected in varying degrees of freedom across the analyses. Participants remembered their guesses on .25 (*SD* = .22) of trials without restudy and on .32 (*SD* = .22) of trials with restudy. The difference between these two rates was significant, *t*(50) = 4.05, *p* < .001, *d* = 0.57, suggesting that guesses were retrieved and strengthened at restudy, providing an opportunity for integration with the restudied to-be-remembered items.

Table 3 presents cued-recall performance depending on restudy opportunity and guess recollection. A 2 (restudy opportunity: restudy, no restudy) x 2 (guess recollection: yes, no) repeated-measures ANOVA revealed a significant main effect of restudy, *F*(1,35) = 19.43, *p* < .001, η_p_^2^ = 0.36. Overall, restudying resulted in higher final accuracy (*M* = *.*37, *SD* = 0.32) compared to the no-restudy condition (*M* = .19, *SD* = .22). Also, there was a significant main effect of guess recollection, *F*(1,35) = 8.62, *p* = .006, η_p_^2^ = .20. Pairs with guesses recollected at test were remembered better, *M* = .37, *SD* = .34, than pairs without correct guess recollection, *M* = .21, *SD* = .21. There was no significant interaction between these factors, *F*(1,35) = 0.28, *p* = .598, η_p_^2^ = .01. We also compared items with correct guess recollection at test to items from the read condition. A 2 (status: recollected guess, read) x 2 (restudy opportunity: restudy, no restudy) repeated-measures ANOVA revealed only a significant main effect of restudy, *F*(1,31) = 24.65, *p* < .001, η_p_^2^ = .44, with higher performance in the restudy, *M* = .43, *SD* = .30, than in the no restudy condition, *M* = .22, *SD* = .23. Neither the main effect of status, nor the interaction was significant, *F*(1,31) = 0.73, *p* = .40, η_p_^2^ = .02, and *F*(1,31) = 0.13, *p* = .72, η_p_^2^ = .004, respectively.

**Table 3:** Cued-recall performance as a function of guess recollection and restudy opportunity in Experiments 2 and 3

	Guess recollection	
Experiment and restudy	Yes	No
Experiment 2		
No restudy	.26 (.04)	.15 (.02)
Restudy	.47 (.05)	.28 (.03)
Experiment 3		
No restudy	.29 (.04)	.16 (.02)
Restudy	.41 (.04)	.30 (.03)

*Note.* Standard errors of the mean are given in parentheses

Experiment 2 replicated the findings of Experiment 1, again showing costs of guessing – relative to reading – in cued recall after studying foreign word translations. Again, this cost was not mitigated by restudy: after restudying the word pairs, reading still remained superior to guessing. These results, which emerged despite the inclusion of the guess detection question at restudy, again undermine the usefulness of guessing as a learning strategy for novel materials such as foreign language vocabulary.

When looking only at the guess condition and comparing items with and without guess recollection, we found a similar pattern of results as Metcalfe and Huelser (2020): target recollection was greater for items for which guesses were remembered rather than forgotten. Also, restudy by itself seemed to increase the chances that one’s guesses would be recollected at test, suggesting that participants at least sometimes were reminded of their guesses during restudy. However, this did not translate into improved performance overall, as even pairs for which guesses were recollected were not remembered better than pairs from the read condition. This pattern leaves open the possibility that differences across pairs for which guesses were versus were not recollected at test reflect a type of an item-selection artifact, by which items that are more likely to be correctly remembered are the same items for which guesses are more likely to be remembered.

Before we can conclude that guessing cannot outperform reading foreign words and their translations – whether one can gain access at test to one’s initial guess or not – it is worth noting that the overall rate of guess recollection in Experiment 2 was only around 28%. This provides an alternative interpretation of the results by which guess recollection does improve performance above the level observed in the read condition, but with the materials that we used it simply occurs too rarely for this difference to be reliably observed. According to this account, the problem with using guessing for learning novel materials is that participants too rarely remember their guesses, even if they engage in restudy when they could be reminded of them. Our main objective in designing Experiment 3 was to make it easier for participants to retrieve their initial guesses and thus increase the overall guess recollection rate. To this end, participants in Experiment 3 had their guesses constrained: they had to begin with the same two letters as the Finnish cue.

## Experiment 3

In the present experiment, we aimed at making guess recollection more prevalent at test. We reasoned that without any knowledge of the to-be-learned Finnish words, participants’ guesses were unlikely to be sensibly related to the cues, and more likely to rely on some random contextual features (e.g., the preceding study item). Such guesses would be very difficult to remember when contextual features change between study and restudy and then between restudy and test. Without access to semantic features of the Finnish words, the easiest strategy for generating guesses that could be retrieved later would be to rely on phonetic information embedded in a cue. Thus, in Experiment 3 we explicitly instructed participants to generate their guesses based on the first two letters of the cue. In this case, the generated words would not so much be guesses at the meaning of the cue – participants could quickly discern that the Polish translations do not start with the same two letters as their Finnish counterparts – but still could serve as potential mediators, similar to those used in the keyword technique for foreign vocabulary acquisition (Lawson & Hogben, 1998). We were again interested in whether recollecting such self-generated episodic mediators at test would support performance over and above performance in the read condition.

### Method

#### Participants

Sixty SWPS University students (11 male; age range 20–52 years, mean: 29.5) took part in the experiment in exchange for course credit. They were all tested online, in the same way as in Experiment 2. We excluded one participant due to technical difficulties which terminated the experiment, another two participants as they were found to be making notes during the learning phase, another three who failed to provide guesses and maintained floor-level accuracy, and one person who did not understand the instructions. This gave us a final sample of 53 participants.

#### Materials and design

The design was the same as in Experiment 2, and can be seen in Fig. 2. Because in this experiment all guesses had to start with the same two letters as the Finnish cue, we had to replace some our study materials so that all words would satisfy the following conditions: (1) each Finnish word had to start with two letters that could also serve as the initial two letters of a Polish word; (2) each combination of two initial letters had to be unique; and (3) no Polish translation could start with the same two letters as any of the Finnish associates.

#### Procedure

The procedure was modelled on that from Experiment 2, with the following exception. During the learning phase, we instructed our participants to provide guesses starting with the same two letters as the Finnish word. For instance, for the cue PISTE (Finnish for *dot*) its first two letters (PI____) were presented next to it, and the guess had to start with them. The sole aim of this manipulation was to increase the guess recollection rate – we assumed that the two initial letters should constitute a good cue to remind oneself of the initial guess.

### Results and discussion

Participants provided no correct guesses, simply because they were constrained by the first two letters of the cue, which were never the same as for the target. Therefore, no items were excluded from the analysis on this basis.

#### Cued recall

Table 2 presents mean cued-recall performance across conditions. A 2 (learning condition: read, guess) x 2 (restudy opportunity: restudy, no restudy) repeated-measures ANOVA revealed a significant main effect of restudy, *F*(1,52) = 84.23, *p* < .001, η_p_^2^ = .62. Overall, restudying items produced better cued-recall performance, *M* = .35, *SD* = .22, compared to items deprived of the restudy opportunity, *M* = .18, *SD* = .17. The main effect of learning condition was not significant, *F*(1,52) = 3.47, *p* = .068, η_p_^2^ = .06, even though there was again a trend toward better performance in the read than in the guess condition, *M* = .28, *SD* = .23, and *M* = .25, *SD* = .19, respectively. The interaction of the two factors was not significant, *F*(1,52) = 0.01, *p* = .917, η_p_^2^ < .001.

#### Guess detection at restudy

Out of all items from the guess condition, 67% were correctly classified as such at restudy. Cued-recall performance for items with and without guess detection can be seen in Fig. 3. There was no significant difference in final cued-recall performance between items correctly identified at restudy as being from the guess condition compared to those which were incorrectly labelled as read: *t*(49) = 0.22, *p* = .827, *d* = 0.03.^4^ This stands in contrast to our findings from Experiment 2.

#### Guess recollection at test

As in Experiment 2, we compared final cued-recall performance for items with and without successful guess recollection, including only items with guesses typed in during the learning phase. The guess recollection rates were .29 (*SD* = .18) for non-restudied items and .40 (*SD* = .25) for restudied items. This difference was significant, *t*(50) = 4.19, *p* < .001, *d* = 0.59, again suggesting that guesses were spontaneously retrieved during restudy.

Table 3 presents cued-recall performance depending on restudy opportunity and guess recollection. A 2 (restudy opportunity: restudy, no restudy) x 2 (guess recollection: yes, no) repeated-measures ANOVA revealed a significant main effect of restudy, *F*(1,43) = 18.47, *p* < .001, η_p_^2^ = .30. Overall, targets from restudied pairs were retrieved more often, *M* =.35, *SD* = .27, than those from pairs that were not restudied, *M* = .22, *SD* = .25. Also, there was a significant main effect of guess recollection, *F*(1,43) = 8.41, *p* = .006, η_p_^2^ = .16. Targets from pairs for which guesses were recollected were retrieved more often, *M* = .35, *SD* = .31, than items with unrecalled guesses, *M* =.23, *SD* = .21. The interaction between these factors was not significant, *F*(1,43) = 0.28, *p* = .598, η_p_^2^ = .01. Finally, we compared memory for items with correct guess recollection at test and those from the read condition with a 2 (status: recollected guess, read) x 2 (restudy opportunity: restudy, no restudy) ANOVA. The results were the same as in Experiment 2. Only the main effect of restudy was significant, *F*(1,44) = 26.78, *p* < .001, η_p_^2^ = .38, with higher performance after restudy, *M* = .39, *SD* = .27, compared to no restudy, *M* = .24, *SD* = .26. Neither the main effect of status, nor the interaction was significant, *F*(1,44) = 2.95, *p* = .09, η_p_^2^ = .06, and *F*(1,44) = 0.67, *p* = .42, η_p_^2^ = .02, respectively.

Experiment 3 replicated the previous two experiments in failing to show a benefit of guessing on cued recall after restudying foreign language translations. In this experiment, the main effect of learning condition was not significant but the numerical trend was consistent with the overall benefit of reading found in previous experiments. Thus, guessing was once again not superior to reading, even after restudying the material and – consistent with Experiment 2 – even after correctly recollecting the guesses at test.

It has to be noted that we did not manage to substantially increase the overall guess recall rate by providing first two letters of a word. In Experiment 2, participants remembered .32 of their guesses after restudy, while the same proportion in the present study was .40, which was still not even half of the initially generated guesses. Thus, it is still possible that guessing would be an effective learning strategy if it were implemented in such a way that participants would remember the vast majority of their guesses at test. The problem with this argument is, however, that remembering associates of unfamiliar foreign words is the exact difficulty that guessing is supposed to ameliorate. If participants find it difficult to associate translations with their respective foreign words, it is perhaps unsurprising that their guesses are also difficult to associate and thus not likely to be later retrieved. Overall, thus, guessing seems to be suboptimal for learning foreign vocabulary – a problem which restudy does not serve to remedy – because guesses are not likely to serve as good episodic mediators, possibly because they are rarely recollected at test in the first place.

## Experiment 4

All experiments reported so far show a clear pattern of results. Adding a restudy phase improved cued-recall performance, but this improvement was similar regardless of whether the pairs were previously read or required guessing. The question remains, however, of why exactly learning condition and restudy did not interact in our study despite previous studies showing that better item memory for words constituting a pair should facilitate creation of associations between those items (Reder et al., 2013, 2016; Vaughn et al., 2016). It is worth noting that the benefits of item memory have been previously observed for the creation of *novel* associations, which deviates from the situation documented in the present study, where at restudy participants are asked to learn associations already introduced in the initial reading/guessing phase. This difference may be crucial if one considers that not only does guessing strengthen item memory, as revealed by recognition results in Experiment 1 (see also Seabrooke et al., 2019), but it also clearly impairs associative memory, as revealed by worse cued-recall performance than in the reading condition. It is possible that while creating novel associations at restudy is indeed augmented by increased item memory, this effect is counteracted by the fact that already encoded associations are more prevalent or stronger in the read condition. In this formulation, more effective creation of novel associations in the guess condition and strengthening of the existing associations in the read condition cancel each other out, leading to equal benefits accruing from restudy.

This hypothesis leads to a straightforward prediction that differentiated benefits of restudy would be observed if *both* item and associative memory after initial study were stronger in the guess than in the read condition. While such a pattern does not occur with the foreign vocabulary used for study in Experiments 1–3, it is exactly what has been observed for weakly related associates, where stronger associative memory has been documented by better cued recall in the guess condition (Kornell et al., 2009) and stronger item memory has been documented by better recall of targets with extra-list cues (Zawadzka & Hanczakowski, 2019). Thus, in Experiment 4 we assessed the effects of restudy across read and guess conditions using weakly related word pairs as study materials. If equal effectiveness of restudy observed thus far has been due to a trade-off between more effective creation of novel associations in the guess condition and more effective strengthening of existing associations in the read condition, then with current materials we would expect both of these processes to be more effective in the guess condition, leading to larger effects of restudy after guessing than reading.

### Participants

Sixty-one students of the SWPS University (13 male**;** age range 19–47 years, mean: 27.6) took part in the experiment in exchange for course credit. We planned to recruit 60 participants, as in previous experiments, but we tested all people who signed up for the study.

### Materials and design

Experiment 4 was based on Experiment 1, with the following differences. We changed the materials from Finnish-Polish translations to weakly related word pairs. As there are no association norms in Polish, we chose pairs of words with an average forward association strength of .05 from association norms (Nelson et al., 2004) and translated them into Polish. As in the previous experiments, we used 64 word pairs for the experiment proper (with two additional ones used in a training phase). In line with Experiments 2 and 3, a cued-recall test was administered for all pairs. Also, there was a single learning and testing phase instead of two study-test blocks. This was done to ensure adequate performance levels as after pilot testing we observed that in the two-block procedure performance after restudy was at ceiling. To further reduce performance, we implemented a 20-min delay before the cued-recall test. During this time, participants completed an unrelated experiment.

### Procedure

Participants were presented with a single list of 64 weakly related word pairs for study. Each pair was presented for 13 s in the read condition. In the guess condition each cue was first presented alone for eight seconds, during which time participants had to guess what the target might be; after that time, they were presented with the full pair for five seconds. For half of the pairs from each condition, a restudy phase followed during which full pairs were presented for 5 s. After the restudy phase, participants completed an unrelated experiment, which took approximately 20 min. Finally, all cues were presented one by one and participants had to type in their corresponding targets or skip to the next pair if no target was retrieved.

#### Results and discussion

In the learning phase, 36 paired associates were guessed correctly, which constituted 1.8% of all trials. These trials were removed from subsequent analyses.

### Cued recall

Table 4 presents mean cued-recall performance across conditions. A 2 (learning condition: read, guess) x 2 (restudy opportunity: restudy, no restudy) repeated-measures ANOVA revealed a significant main effect of learning condition, *F*(1,60) = 35.09, *p* < .001, η_p_^2^ = .37. Overall, guessing improved cued-recall performance compared to reading, *M* = .69, *SD* = .23, and *M* = .58, *SD* = .28, respectively. Also, there was a significant main effect of restudy, *F*(1,60) = 70.04, *p* < .001, η_p_^2^ = .54. Restudying pairs resulted in higher performance *– M* = .72, *SD* = .25 – compared to *M* = .55, *SD* = .25, for not restudied items. The interaction between the two conditions was not significant, *F*(1,60) = 1.83, *p* = .182, η_p_^2^ = .03, and if anything, the benefits of restudy were numerically larger in the read than in the guess condition.

**Table 4:** Cued-recall performance as a function of learning condition and restudy opportunity in Experiment 4

	Learning condition	
Restudy opportunity	Guess	Read
No Restudy	.62 (.03)	.48 (.03)
Restudy	.76 (.03)	.68 (.03)

*Note.* Standard errors of the mean are given in parentheses

In accordance with previous research, guessing outperformed reading when weakly related word pairs were used as stimuli. This indicates that this time associative memory after the initial study was stronger in the guess than in the read condition. Still, the overall pattern of results from our first three experiments was replicated in Experiment 4 as well, with significant main effects of restudy and learning condition but no interaction between the two. This shows that no matter whether reading or guessing leads to better cued-recall performance, these benefits persist even after an additional learning session. This result stands in contradiction to the idea that the results of Experiments 1–3 were due to item and associative information differentially supporting encoding at restudy across guess and read conditions. Of course, it is worth pointing out that the theoretical implications one can draw from the lack of an ordinal interaction are necessarily limited (see Wagenmakers et al., 2012). The effectiveness of restudy in the guess condition could be undermined by the fact that a higher proportion of pairs had been already learned after guessing than after reading. Still, on an empirical side, these results confirm that the initial patterns of memory after guessing and reading are not strongly related to how effective a restudy opportunity is likely to be.

## General discussion

Is guessing a good strategy for studying foreign vocabulary? Based on the present experiments, it seems not. Throughout a series of three experiments using Finnish-Polish translations as study materials we consistently showed that guessing leads to worse cued-recall performance than reading. This was regardless of whether the translations were studied once or twice, contrary to our prediction that guessing should facilitate encoding of foreign word-to-translation associations at restudy.

Experiment 1 demonstrated that despite the fact that guessing did strengthen the targets, as evidenced by better recognition performance, reading was a more effective strategy as reflected in cued-recall results. This chimes with the findings of Seabrooke et al. (2019), who were the first to demonstrate that guessing might not be the best strategy for foreign vocabulary acquisition. Importantly, this pattern held regardless of whether translations were studied once or restudied. Experiments 2 and 3 extended this finding by showing that even when participants remembered their guesses – and so could in principle use them as episodic mediators for target retrieval – guessing still was unable to outperform reading as a learning strategy.

To further strengthen this conclusion, we performed a Bayesian repeated-measures ANOVA on the combined data from Experiments 1–3. This analysis (total *N* = 162) showed that the evidence for the main effects on their own was extreme: BF_(inclusion)_ = 157,153.26 for the main effect of learning condition, and BF_(inclusion)_ = 8.601e+64 for the main effect of restudy. In contrast, there was moderate evidence against the interaction of learning condition and restudy, BF_(inclusion)_ = 0.126. Therefore, we can safely conclude that while it matters what learning strategy people use and whether they restudy the materials, the two factors are independent of one another. This stands in contrast to our prediction that strengthened targets should be easier to associate with their foreign equivalents when a restudy opportunity is available.

To assess the generalizability of the findings from Experiments 1–3 regarding the ineffectiveness of restudy in ameliorating performance differences across the reading and guessing conditions, we conducted Experiment 4 with weakly related word pairs as study materials. With these materials, an advantage of guessing over reading in measures of both associative (e.g., Grimaldi & Karpicke, 2012; Huelser & Metcalfe, 2012; Kornell et al., 2009) and target memory (Zawadzka & Hanczakowski, 2019) is robustly obtained. In line with the many previous observations, Experiment 4 replicated the guessing benefit in cued-recall performance. However, we again failed to observe an interaction between the learning condition and restudy. Therefore, in a series of four experiments we confirmed that both costs (Experiments 1–3) and benefits (Experiment 4) of guessing are not easily modified by adding a restudy opportunity.

The take-home message from this study is thus threefold. First, we have consistently shown that it is the initial encounter with the to-be-learned material that determines the overall effectiveness of learning. Restudy further aids learning and does so to a large extent, but does not seem to modify the patterns obtained during the initial learning phase. This is not to say that relearning is never effective in modifying the initial learning patterns. For example, Rawson and Dunlosky (2014) documented how relearning negates the benefits initially accruing from spaced learning. However, these authors used initial learning to criterion and multiple relearning sessions. It is likely that limited relearning in a single study session has markedly less pronounced effects, as shown here. This observation is of high practical importance given the limited time and effort people often spend in the learning process. The fact that the patterns of performance resulting from this first study opportunity are not modifiable by an additional session of study speaks to the importance of choosing an appropriate strategy for the initial learning. Given that the first encounter with study materials is likely to happen in organized educational settings such as classrooms, our findings underscore the role of appropriate strategy choice on the part of educators.

Second, our data provide a clear demonstration that guessing a translation of a foreign word can actually impair memory compared to reading the foreign word-translation pair outright. The combined Bayesian analysis of Experiments 1–3 provided extreme evidence for a difference between reading and guessing. The results published so far were less decisive: across the experiments published by Seabrooke et al. (2019), only one – Experiment 5 – revealed a decrease in performance in the group that had to guess the translation of an Euskara word rather than read it. Interestingly, this was their only experiment that focused on foreign vocabulary translations, as the remaining experiments used rare English words as study materials. It would thus seem that guessing might be a particularly harmful strategy for learning a foreign language.

Finally, the lack of an interaction between learning condition and restudy is of interest from a theoretical point of view. Despite research showing that greater familiarity of individual components of to-be-learned materials should aid in associating these components (Gagnepain et al., 2008; Reder et al., 2016), here we have failed to observe such a pattern. Regardless of the materials used, it did not appear to be easier for participants to associate stronger targets with cues at restudy. It is worth noting that recent years saw a surge in studies assessing the role of item memory in encoding associations, and conclusions from this literature are not clear, with some studies showing better encoding of associations for stronger items (e.g., Greve et al., 2017; Poppenk & Norman, 2012), while others showed the opposite pattern (Kim et al., 2012). Recent work by Lee et al. (2020) suggested a modulating role of pre-experimental familiarity of study materials, with novel stimuli yielding a benefit of increased item strength for association formation and familiar stimuli yielding a cost. However, in Experiments 1–3 we used stimuli that were completely novel to participants and in Experiment 4 we used stimuli – words – that were familiar, and the results were the same, with equal associative memory across conditions differing in item strength before restudy. This consistent null pattern does not fit any theories that try to account for either the benefits of the costs observed in previous studies. This issue clearly awaits further research.

It should also be noted that our results do not follow the patterns generally found in the test-potentiated learning literature, as memory for pairs initially requiring guessing did not benefit from restudy to a greater extent than memory for read pairs. There are three differences between our paradigm and that commonly used in research on test-potentiated learning (e.g., Arnold & McDermott, 2013a, 2013b; Izawa, 1971) that might have been responsible for those discrepancies. First, here guessing was applied during the first encounter with the studied pairs, while studies on test-potentiated learning include an initial study phase that entails reading and only then is the presence of retrieval attempts manipulated. Second, those procedures usually employ multiple testing cycles rather than a single one used here. Finally, there remains a question of how much guessing is based on retrieval of information from memory. It can be argued that the less specific information is present in the cue, the less retrieval there will be: for example, guessing answers to trivia questions (Kornell, 2014) is more likely to draw from one’s semantic memory than attempting to guess which associate of a noun will be the target, or what the translation of a completely unknown foreign word might be. Thus, the failure to obtain the increased benefits of restudy in the guessing condition could perhaps help establish boundary conditions for test-potentiated learning.

Altogether, the results documented in the present study do not seem to fit well with theoretical considerations present in the learning literature. This is not to argue that these results are inconsistent with the extant theories, as there are many procedural differences between our study and studies designed to assess the influence of item strength on encoding associations or studies on test-potentiated learning. The point is rather that theoretical mechanisms are commonly revealed in paradigms specifically tailored for highlighting subtle mechanisms of memory functioning. These mechanisms, although necessarily operating whenever memory is engaged, may still be not potent enough to strongly influence learning as it occurs in more applied settings, including foreign vocabulary acquisition investigated here.

In conclusion, our research challenges a recommendation made by Potts and Shanks (2014) of using guessing as an effective strategy for learning the meaning of novel words. Although we replicated the benefit of guessing over reading in a recognition test, we consistently demonstrated guessing inferiority in a more appropriate cued-recall test, replicating recent results obtained by Seabrooke et al. (2019). Crucially, this pattern of costs was not ameliorated by the opportunity to restudy materials, with restudy benefiting performance but preserving the overall pattern of differences across learning strategies, both in situations in which guessing yielded costs and benefits to memory retention. This persistence of the effects of the initial learning strategy points to the crucial role of how information is acquired when it is first presented for study.

## Appendix 1

### Conditional analyses of guess detection at test

#### Experiment 2

We compared guess detection rates for restudied and not restudied items. The rates were 51% and 57% for not restudied and restudied items, respectively. There was a significant difference between the conditions, *t*(49) = 2.24, *p* = .029, *d* = 0.32.

Appendix Table 5 presents cued-recall performance depending on restudy opportunity and guess detection. A repeated-measures ANOVA revealed a main effect of restudy, *F*(1,50) = 30.97, *p* < .001, η_p_^2^ = .38. Restudying pairs resulted in the overall accuracy of .29 (*SD* = .26) compared to the accuracy rate of .15 (*SD* = 0.19) for not restudied pairs. Also, there was a significant main effect of guess detection, *F*(1,50) = 24.74, *p* < .001, η_p_^2^ = .33. Pairs correctly identified at test as initially guessed were recalled at the rate of .29 (*SD* = .25) compared to .15 (*SD* = 0.20) for incorrectly identified items. There was no significant interaction between these factors, *F*(1,50) = 3.23, *p* = .078, η_p_^2^ = .06.

#### Experiment 3

We compared guess detection rates for restudied and not restudied items. The rates were 43% and 56% for not restudied and restudied items, respectively. There was a significant difference between the conditions, *t*(51) = 4.97, *p* < .001, *d* = 0.69.

Appendix Table 5 presents cued-recall performance depending on restudy opportunity and guess detection. As in Experiment 2, a repeated-measures ANOVA revealed a main effect of restudy, *F*(1,51) = 35.51, *p* < .001, η_p_^2^ = .41. Overall, restudy benefitted final cued-recall performance compared to a single study opportunity (*M* = .32, *SD* = .26, and *M* = .17, *SD* = .18, respectively). Also, there was a main effect of guess detection, *F*(1,50) = 9.38, *p* = .004, η_p_^2^ = .16. Pairs which were correctly identified at the final tests as being guessed during the learning phase were recalled at the rate of .29 (*SD* = .24) compared to .21 (*SD* = .23) for pairs which were incorrectly identified. There was no significant interaction, *F*(1,50) = 0.47, *p* = .496, η_p_^2^ = .01.

**Table 5:** Cued-recall performance as a function of guess detection and restudy opportunity in Experiments 2 and 3

	Guess detection	
Experiment and condition	Yes	No
Experiment 2		
No restudy	.20 (.02)	.10 (.02)
Restudy	.37 (.04)	.20 (.03)
Experiment 3		
No restudy	.22 (.03)	.13 (.02)
Restudy	.35 (.03)	.29 (.04)

*Note.* Standard errors of the mean are given in parentheses

## Footnotes

1 Participants failed to provide guesses on 6% of trials in Experiment 1, 13% in Experiment 2, 24% in Experiment 3, and 9% in Experiment 4.

2 All cued-recall analyses reported for Experiments 1–3 were performed on the combined data from block-1 and block-2 tests. We additionally performed the analyses described above with an additional factor of block. In none of the experiments did block enter into any interaction, but the main effect of block was always significant, with higher performance in block 2 than block 1, which is likely due to the forward testing effect for learning new materials (Chan et al., 2018; Yang et al., 2021).

3 See Appendix 1 for an analysis conditionalized on whether participants correctly indicated the learning condition on the final test.

4 The analysis conditionalized on whether participants correctly indicated the learning condition measured at test can be found in Appendix 1.
